# Eosinophil Cell Count Predicts Mortality in the Intensive Care Unit after Return of Spontaneous Circulation

**DOI:** 10.5041/RMMJ.10458

**Published:** 2022-01-27

**Authors:** İlhan Korkmaz, Yusuf Kenan Tekin, Gülaçan Tekin, Erdal Demirtaş, Sefa Yurtbay, Naim Nur

**Affiliations:** 1 Department of Emergency Medicine, Sivas Cumhuriyet University, Faculty of Medicine, Sivas, Turkey; 2 Department of Cardiology, Sivas Cumhuriyet University, Faculty of Medicine, Sivas, Turkey; 3 Department of Emergency Medicine, Dokuz Eylül University, Faculty of Medicine, İzmir, Turkey; 4 Department of Family Medicine, Sivas Cumhuriyet University, Faculty of Medicine, Sivas, Turkey

**Keywords:** Cardiac arrest, eosinophil, prognosis, resuscitation

## Abstract

**Background:**

Eosinophils constitute 1%–5% of peripheral blood leukocytes, less in the presence of acute infections (referred to as eosinopenia). Studies indicate that eosinopenia can be used as a prognostic predictor for chronic obstructive pulmonary disease exacerbation, sepsis, or acute myocardial infarction disease. There are only a few studies about predicting mortality in emergency departments and intensive care units (ICUs). Prognostic studies about patients in ICUs are generally carried out using different scoring systems. We aimed to analyze if the eosinophil count can estimate the prognosis among non-traumatic patients who underwent cardiopulmonary resuscitation and were hospitalized in ICU thereafter.

**Methods:**

The data were evaluated of 865 non-traumatic adult patients (>18 years of age) who were admitted with cardiopulmonary arrest or developed cardiopulmonary arrest during clinical follow-ups. Admission venous blood sample tests, complete blood count, and biochemical laboratory results were recorded. Arterial blood gas results were also evaluated. The mean results of the recorded laboratory results were compared between the surviving and non-surviving patients groups.

**Results:**

There was a significant difference between the two groups in regard to platelet, eosinophil count, pH, PaO_2_, SaO_2_, and HCO_3_^−^ (*P*<0.001 for all). In the multiple linear regression analysis, eosinophil counts were found to be an independent factor (odds ratio=0.03, 95% confidence interval 0.33–0.56, *P*<0.001) associated with the mortality after cardiopulmonary resuscitation.

**Conclusion:**

Because admission eosinophil counts can be measured easily, they are inexpensive biomarkers that can be used for predicting the prognosis among the patients who have return of spontaneous circulation and are treated in ICUs.

## INTRODUCTION

Eosinophils were first described by Paul Ehrlich in 1879.^1^ Constituting 1%–5% of the peripheral blood leukocytes, eosinophils represent up to 6% of the nucleated cells in bone marrow. Zappert first described the reduction of eosinophil counts during acute infections in 1893.^2^ To date, eosinophils have mainly been known as multifunctional pro-inflammatory white blood cells involved in allergic disorder pathogenesis, and have a primary role in parasitic infections.^1^–^4^

The main pathway inhibiting the exit of eosinophils from bone marrow is not defined clearly in inflammatory states. Cayrol and Girard determined that acute stress and organ infarct can stimulate adrenaline and glucocorticoid production by the adrenal glands, leading to eosinopenia by apoptosis. There are multiple factors thought to cause eosinopenia, such as: eosinophilopoiesis blockage, chemokine receptor/adhesion factor reduction, and/or direct eosinophil apoptosis induced by type 1 interferons.^5^

Use of eosinopenia as a prognostic biomarker has been analyzed in chronic obstructive pulmonary disease exacerbation, sepsis, and acute myocardial infarction.^6^–^8^ There are only a few studies regarding the use of eosinopenia to predict mortality in emergency departments and intensive care units (ICUs). Prognostic studies on ICU patients are carried out using various scoring systems.^9^ Some studies have described different types of new biomarkers, including plasma Mg, high-mobility group protein B1, and urokinase-type plasminogen activator receptor, in the ongoing search for better tools for prognosis of mortality.^10^–^12^

However, eosinophil count would be a more attractive biomarker due to its availability, low cost, and minimum delay between taking blood samples and obtaining results, compared to standard scoring systems and other suggested biomarkers. This study therefore was aimed at determining whether or not eosinophil count can be used for prognostic estimates in non-traumatic patients who have undergone cardiopulmonary resuscitation (CPR) and were subsequently hospitalized in an ICU.

## MATERIALS AND METHODS

This research was approved by the Cumhuriyet University Medicine Faculty Human Ethics Committee (2019-10/02). A retrospective analysis was performed of 865 non-traumatic adult patients (>18 years of age), who had been admitted with cardiopulmonary arrest, or experienced cardiopulmonary arrest during clinical follow-ups in the emergency services, and successfully resuscitated and hospitalized under anesthesia in the ICU between January 2010 and June 2019. Excluded from the study were 59 patients with a previous history of beta-blocker use, pre-hospital epinephrine administration, sepsis, cytotoxic-steroid use, or immunodeficiency. Hence the data of 806 patients were included in this study.

### Laboratory Analyses

Blood samples were obtained on admission. White blood cell and eosinophil cell counts were determined with a BC-6800 analyzer (Mindray, Toshiba, Tokyo, Japan). Other biochemical laboratory tests were performed with an Image 800 analyzer (Beckman Coulter, Yokohama, Japan) using the accompanying manufacturer’s kit via a fully automated nephelometric method.

All data analyses were performed using SPSS (version 23.0) software (SPSS Inc., Chicago, IL, USA) licensed by Sivas Cumhuriyet University. Descriptive statistics were expressed as a means, standard deviations, and frequencies. Student’s *t*-test was used to compare the mean laboratory values after homogeneity of variance had been tested by Levene’s test of equality of variances. Receiver-operating characteristics (ROC) curve and the area under the curve were calculated for both eosinophil counts and percentage at admission. The best eosinophil cut-off value was chosen using Youden’s index. Multiple linear regression was performed to assess independent factors associated with the mortality after cardiopulmonary resuscitation. A *P* value of less than 0.05 was considered to be significant.

## RESULTS

Of the 806 patients included in the study, 475 (58.6%) were male, 331 (41.4%) were female, and the mean age was 68.9±13.9 years. The period of time between resuscitation and admission of the patients to the ICU was 30.1±8.2 minutes.

Statistical differences were noted between the surviving and non-surviving groups in terms of platelets, eosinophil count, eosinophil percentage, pH, PaO_2_, SaO_2_, and HCO_3_^−^ (Table 1).

**Table 1:** Laboratory Results Compared According to the Survivor and Non-survivor Groups.

Variables	Survivors(*n*=49, mean±SD)	Non-survivors(*n*=757, mean±SD)	*P* Value
White blood cells (10^3^/μL)	20.47±8.34	18.46±10.52	0.18
Hemoglobin (g/dL)	13.60±2.54	12.86±2.67	0.05
Platelets (10^3^/μL)	354.17±171.19	245.50±122.95	0.001
Eosinophil count (10^3^/μL)	0.41±0.28	0.14±0.16	0.001
Eosinophil (%)	4.42±2.97	1.35±2.04	0.001
pH	7.44±0.50	7.21±0.37	0.001
PaCO_2_ (mmHg)	61.01±21.42	58.86±27.03	0.55
PaO_2_ (mmHg)	160.11±85.54	106.71±76.21	0.001
SaO_2_ (%)	80.01±27.88	97.15±5.05	0.001
HCO_3_^−^ (meq/L)	28.90±7.36	19.31±9.30	0.001
Blood urea nitrogen (mg/dL)	53.84±32.33	56.56±37.91	0.61
Creatinine (mg/dL)	2.76±3.15	2.80±2.13	0.90

**Figure 1:**
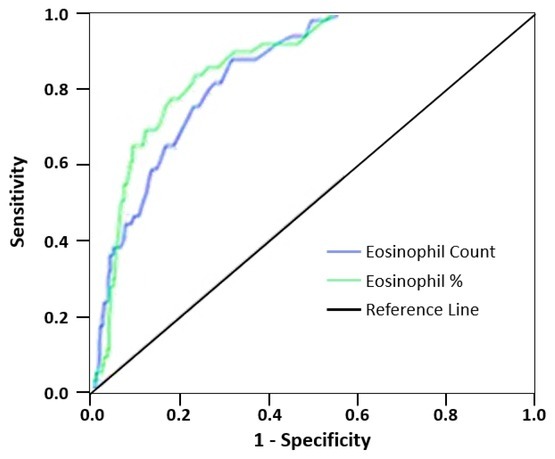
ROC Curve of Eosinophil Count and Eosinophil Percentage at the time of Admission to the Emergency Department Discriminates between Survivors and Non-survivors.

The optimum cut-off value for both eosinophil and eosinophil percentage in this study were deter-determined by ROC analysis. The optimum eosinophil cut-off value was 0.26 (area under the curve [AUC], 0.850; 95% confidence interval [CI] 0.806–0.894; sensitivity, 83%; specificity, 67%). For eosinophil percentage, the cut-off value was 2.75 (AUC, 0.872; 95% CI 0.830–0.913; sensitivity, 87%; specificity, 71%) (Table 2 and Figure 1).

**Table 2:** Cut-off Value, Sensitivity, and Specificity of Esinophil Cell Count for the Mortality Rate.

Parameter	Eosinophil Count	Eosinophil %
Cut-off value	0.26	2.75
Sensitivity	0.83	0.87
Specificity	0.67	0.71
AUC (95% CI)	0.850 (0.806–0.894)	0.872 (0.830–0.913)

Logistic regression analysis showed that white blood cell, platelet, and eosinophil counts were independently associated with mortality after cardiopulmonary resuscitation (Table 3).

**Table 3:** Multiple Linear Regression for Predicting the Mortality after Cardiopulmonary Resuscitation.

Independent Parameters	95.0% Confidence Interval for Beta		Beta	*P* Value
	Lower Limit	Upper Limit		
White blood cell (10^3^/L)	0.325	0.556	0.259	0.000
Platelet (10^3^/μL)	0.000	0.001	−0.246	0.000
HCO_3_^−^	0.000	0.006	−0.064	0.092
Neutrophil (10^3^/μL)	−0.003	0.003	−0.269	0.909
Eosinophil (10^3^/μL)	0.325	0.556	0.028	0.000
PaCO_2_	−0.001	0.000	0.056	0.226
PaO_2_	0.000	0.000	−0.140	0.493
pH	−0.066	0.121	0.070	0.566
SaO_2_ (%)	−0.001	0.001	0.011	0.760

*R^2^* adjusted=0.195.

## DISCUSSION

Our study highlights the importance of eosinopenia as a predictor of mortality among patients admitted to the emergency service and hospitalized in ICUs. The results show that non-survivors had significantly decreased eosinophil counts. Mortality was also predicted with a high sensitivity by the admission eosinophil count in the emergency department. Multiple linear regression demonstrates that the eosinophil count predicted mortality more strongly than other laboratory markers.

Eosinophils mainly play a role in the pathogenesis of allergic, hematologic, parasitic, and some skin diseases. The granulocyte-macrophage colony-stimulating factor (GM-CSF) and interleukins (IL) IL3 and IL5 are the main myeloid precursors for the development and maturation of eosinophil cells in the bone marrow. The mechanism of eosinopenia is also associated with other chemotactics such as complement 5a (C5a), which causes intravascular migration, tissue migration, and intravascular cell destruction. Cytokine inhibition plays a role in the bone marrow for the production and release of eosinophils.^13^

Bass and co-workers confirmed that eosinophil counts decrease in acute inflammatory diseases and the cell count returns to normal values on recovery.^14^,^15^ Different inflammatory biomarkers are used to predict mortality. A systematic review by Zhang and Ni showed that only the late C-reactive protein concentration should be used to identify patients at risk of death, and that it could not be used as a marker in emergency departments.^16^

No study has investigated the prognostic value of eosinophil count for patients admitted to the ICU after cardiopulmonary arrest. Davido et al. evaluated increased eosinophil count as a marker among patients with bacterial infection to assess whether or not a patient was receiving the appropriate antibiotic. They noted a significantly increased eosinophil count among patients by day 1 after receiving effective antimicrobial therapy.^17^

The relationship between eosinophil count and infection has also been evaluated in critical patients. Salem et al. looked at eosinopenia as a potential predictor for sepsis diagnosis and mortality in critically ill patients admitted to ICU. They found that admission eosinophil counts of <50 cells/mm^3^ could be used as a predictor for sepsis diagnosis in ICU patients, although the admission eosinophil sensitivity and specificity did not predict mortality.^18^

In another study by Swaminathan et al., the absolute eosinophil count was a reliable marker of mortality in patients with perforative peritonitis, as the values decreased in patients who did not survive.^19^

Acute exacerbation of chronic obstructive pulmonary disease is frequently seen in emergency departments. The patient’s clinical severity is primarily determined based on arterial blood gas tests, which are invasive. Rahimi-Rad et al. examined the use of eosinopenia as a biomarker for outcomes. They found that the in-hospital mortality rate among eosinopenic patients was significantly higher than non-eosinopenic patients and concluded that eosinopenia could be a useful, easy-to-measure, and inexpensive biomarker for predicting prognosis.^7^ In our study, the mean SaO_2_ of the survival group was lower than that of the non-surviving group. Although this result seems contradictory, the difference may have been due to how their airways were maintained: the non-surviving group with higher oxygen values received more effective prehospital intubation and advanced airway applications as compared to the survival group, who were treated mostly by Ambu mask or nasal oxygen in the prehospital period.

Intracerebral hemorrhage is a disease with high mortality and morbidity rates. In a study by Goswami et al., low Glasgow coma scale (GCS) scores, bilateral limb weakness, high blood pressure, gaze palsies, pupillary abnormalities, hematoma volume ≥30 mL, and midline shift were found to be prognostic factors associated with bad outcomes.^20^ However, admission eosinopenia or eosinophil count/percentage has been found to be a simple prognostic laboratory biomarker for stroke patients. Bolayir et al. evaluated 296 patients with intracerebral hemorrhage, and the eosinophil count and percentage of non-surviving patients decreased significantly as compared to the surviving patients’ records.^21^

Eosinophil counts have also been used to determine outcomes after surgery. In the study of adults undergoing non-cardiac vascular surgery, patients in the eosinopenia group had a higher mortality rate within 90 days, with an OR of 1.97 (95% CI 1.42, 2.73; *P*<0.001) relative to patients with a normal absolute eosinophil count.^22^

In another surgical study, Shivani et al. tested the efficacy of the eosinophil count as a predictor for mortality among patients treated in the surgical ICU. They found that most of the patients who died had an absolute eosinophil count <40/mm^3^ and suggested that patients with eosinopenia paired with a positive blood culture had a higher mortality rate.^23^

Abidi et al. evaluated the variations in eosinophil count from admission to day 7 to predict the 28-day mortality among surviving and non-surviving patients in the ICU. They found that the absolute eosinophil count in non-survivors decreased and remained lower compared with the surviving group, indicating that admission eosinophil cell count could be used as a marker for disease severity of patients hospitalized in the ICU.^24^

## CONCLUSION

Admission eosinophil counts can be measured easily, and are inexpensive biomarkers that can be used for predicting prognosis among patients who have been resuscitated and transferred for ongoing treatment in ICUs.

## Abbreviations

AUC: area under the curve
C5a: complement 5a
CPR: cardiopulmonary resuscitation
GCS: Glasgow coma scale
GM-CSF: granulocyte-macrophage colony-stimulating factor
HCO_3_^−^: bicarbonate
ICU: intensive care unit
IL3: interleukin 3
IL5: interleukin 5
OR: odds ratio
PaCO_2_: partial pressure of carbon dioxide
PaO_2_: partial pressure of oxygen in arterial blood
pH: power of hydrogen
ROC: receiver-operating characteristics
SaO_2_: partial arterial oxygen saturation
